# Fine mapping and candidate gene analysis of a major QTL for panicle structure in rice

**DOI:** 10.1007/s00299-014-1661-0

**Published:** 2014-07-31

**Authors:** Youlin Peng, Zhenyu Gao, Bin Zhang, Chaolei Liu, Jie Xu, Banpu Ruan, Jiang Hu, Guojun Dong, Longbiao Guo, Guohua Liang, Qian Qian

**Affiliations:** 1 Key Laboratory of Plant Functional Genomics of the Ministry of Education, Jiangsu Key Laboratory of Crop Genetics and Physiology College of Agriculture, Yangzhou University, Yangzhou, 225009 China; 2 State Key Laboratory of Rice Biology, China National Rice Research Institute, Chinese Academy of Agricultural Sciences, Hangzhou, 310006 China

**Keywords:** Rice, Quantitative trait loci, Primary panicle branch number, Fine mapping, Panicle structure

## Abstract

*****Key message***:**

**A gene not only control tiller and plant height, but also regulate panicle structure by QTL dissection in rice.**

**Abstract:**

An ideal panicle structure is important for improvement of plant architecture and rice yield. In this study, using recombinant inbred lines (RILs) of PA64s and 93-11, we identified a quantitative trait locus (QTL), designated *qPPB3* for primary panicle branch number. With a BC_3_F_2_ population derived from a backcross between a resequenced RIL carrying PA64s allele and 93-11, *qPPB3* was fine mapped to a 34.6-kb genomic region. Gene prediction analysis identified four putative genes, among which Os03g0203200, a previously reported gene for plant height and tiller number, *Dwarf 88* (*D88*)/*Dwarf 14* (*D14*), had three nucleotide substitutions in 93-11 compared with PA64s. The T to G substitution resulted in one amino acid change from valine in 93-11 to glycine in PA64s. Real-time PCR analysis showed expression level of *D88* was higher in 93-11 than PA64s. The expression of *APO1* and *IPA1* increased, while *GN1a* and *DST* decreased in 93-11 compared with PA64s. Therefore, *D88/D14* is not only a key regulator for branching, but also affects panicle structure.

**Electronic supplementary material:**

The online version of this article (doi:10.1007/s00299-014-1661-0) contains supplementary material, which is available to authorized users.

## Introduction

Plant architecture is very important for improving rice yield, grain quality, resistance to multiple biotic and abiotic stresses, and nutrient utilization efficiency. Panicle structure, belonging to plant architecture, is one of the important factors for rice yield. It includes four components: panicle length, primary panicle branch number, secondary panicle branch number, and spikelet number. Several genes that regulate the development of rice panicle branches have been cloned, such as *MOC1* (Li et al. 2003), *LAX1* (Komatsu et al. 2003), *LAX2* (Tabuchi et al. 2011), *GN1a/OsCKX2* (Ashikari et al. 2005), *DST* (Li et al. 2013), *DEP1* (Huang et al. 2009), *SP1* (Li et al. 2009), and *APO1* (Ikeda et al. 2007).

Panicle branches are lateral organs emerged at reproductive stage, and their number affects spikelet number. In some cases, panicle branches are regulated by common mechanisms shared with tiller formation and elongation. *MOC1* and *LAX1* were genes that regulate both tillers and panicle branches. Reduction in tiller number and panicle branch number was discovered in both mutants. Mutation in *LAX2* led to decrease in tiller number. However, the primary panicle branch number remained unchanged (Tabuchi et al. 2011). The near isogenic lines (NILs) carrying *ipa1* displayed reduced tillers and increased panicle branches (Jiao et al. 2010). *DEP1* (Huang et al. 2009) was a major dominant QTL that control panicle branches. The NIL-*dep1* showed more panicle branches, which may result from cell proliferation through *GN1a* (Huang et al. 2009). *APO1*, a pivotal gene to regulate primary panicle branch, controlled the vascular bundle formation and could increase the harvest index and grain yield in rice (Terao et al. 2010).

In this study, QTL analysis of panicle branches was performed with a RIL population derived from an *indica/javonica* cross, and a major QTL (*qPPB3*) for primary panicle branch number was detected on chromosome 3. The region of *qPPB3* was narrowed by map-based cloning strategy with BC_3_F_2_ population derived from a chromosome segment substitution line (CSSL). Based on sequencing and expression analysis, the predicted *ORF* for *qPPB3* encoding a protein of the α/β-fold hydrolase superfamily (D88/D14), was proved to be not only a key regulator for plant shoot branching (Jiang et al. 2013; Arite et al. 2009; Gao et al. 2009), but also effected primary panicle branching.

## Materials and methods

### Mapping population and phenotyping

The core mapping population of 132 LYP9 RILs was randomly chosen from 1,841 RILs derived by single-seed descend from a cross between an elite paternal inbred *Oryza* *sativa*. *indica* cv. 93-11 and the maternal inbred *Oryza sativa ssp*. *javonica* cv. PA64s, a photo-thermo-sensitive male sterile line with a mixed genetic background of *indica*, *javanica*, and *japonica*. The population was developed in the experimental fields at China National Rice Research Institute in Hangzhou, Zhejiang Province, and in Sanya, Hainan Province, China. After 12 generations of self-fertilization, genomic DNA samples of the F_13_ RILs were isolated for genotyping (Gao et al. 2013). Phenotyping was conducted according to Gao et al. (2013).

### Data analysis and QTL detection

All statistical analyses were completed using the SAS (Statistical Analysis System) V8.01. QTL analysis was performed with the MultiQTL1.6 package (http://www.multiqtl.com) using maximum likelihood interval mapping (MIM) based on a permutation test (1,000 permutation, *P* = 0.05) for each dataset to confirm the LOD threshold. It was considered as a major effect QTL when its LOD was larger than 2.5. QTLs were named according to McCouch (Mccouch et al. 1997).

### Acquisition of CSSL involving *qPPB3*

To develop a CSSL containing *qPPB3*, a line of RILs with PA64s’ genotype in the *qPPB3* region was selected to backcross with recurrent parent 93-11. Two STS markers P1 and P2 (Table 1) were used for marker-assisted selection (MAS) of each generation in the segregating progenies. As a result, a BC_3_F_1_ line GH18, with 93-11 genetic background exhibiting heterozygous across the entire *qPPB3* region, was constructed. After self-crossing, we acquired a BC_3_F_2_ population for fine mapping of *qPPB3*.

**Table 1:** Primers for fine mapping of *qPPB3* and real time PCR

Primer	Forward	Reverse	Experiment
P1	TCGTCATATACTCTTATG	AACCGCACTCTAGATTTGG	Mapping
P2	ACCATGAATCTCAGCTGC	TGCGTTAAGACCGTCCTC	Mapping
P3	TCTCACAAGCATGCACGCTTG	TGTGCTTGCACATGTGCATG	Mapping
P4	ACCTTAGATATTAGATCCAG	TGTGTAAATAGCTAATGTGTG	Mapping
P5	TGTCAGAATCATACATGCACC	AAGCTCATCCCTCAACTCTC	Mapping
P6	TGGCAATCAAGTCAGCATTC	TCTCTGTCCTACTTCTCTAGC	Mapping
P7	TCTTATCTGCTCCCACTTG	TCGATTCGAACCCTCTCGTGC	Mapping
*Actin*	CCATTGGTGCTGAGCGTTT	CGCAGCTTCCATTCCTATGAA	Real-time PCR
*D88*	CGCCTTCGTCGGCCACTC	TCGAACCCGCCGTGGTAGTC	Real-time PCR
*APO1*	GTCATCTGAGTTGGTAGTGTG	CAACAGATCTCATGGCAAG	Real-time PCR
*IPA1*	GGATATGGTGCCAACACATACAG	GACATGGCTGCAGCCTGGTTGTG	Real-time PCR
*GN1a*	TGTCCCTTCTACAATGGTGC	CATCCTGACCTGCTCTTGCT	Real-time PCR
*DST*	GCTACTGCTGGCGTTGGG	GAGATGGTGCTGGTGCGT	Real-time PCR

### Design of fine mapping markers

Primers were designed around *qPPB3* on chromosome 3 using insertions/deletions (InDels) identified between 93-11 and PA64s (Gao et al. 2013) by two software, Primer Premier 5.0 and Oligo 7 (Table 1).

### RNA extraction and real-time PCR analysis

Total RNA was isolated from panicles before booting stage with the micro RNA extraction kit (Axygen). DNase treatment, cDNA synthesis, primer design and SYBR Green I Real-time PCR were carried out as described (Vandesompele et al. 2002) using a ReverTra Ace^®^ qPCR-RT kit (TOYOBA, Japan). Real-time PCR amplification mixtures (10 μl) contained 50 ng template cDNA, 2× SYBR Green PCR Master Mix (10 μl) (Applied Biosystems), and 200 nM forward and reverse primers. Reactions were run on an ABI PRISM^®^ 7900HT Sequence Detector (Applied Biosystems). The relative expression level of each transcript was obtained by comparing to the expression of the *OsACTIN1* gene. Primers for *D88*, *APO1*, *IPA1*, *GN1a*, *DST,* and *Actin* are listed in Table 1.

### Subcellular localization of D88

Full-length cDNA of *D88* was isolated from Nipponbare and sequenced. Then it was inserted into the vector pCAMBIA1302. The D88::GFP fusion was made by inflame fusion of the full-length *D88* cDNA with GFP. Rice transformation was performed by an Agrobacterium-mediated method (Hiei et al. 1994). The root tips of transgenic plants expressing the GFP fusions were observed directly with a microscope (Nikon 90i).

## Results

### Phenotypic performance of panicle branches in parents and RILs

**Fig. 1:**
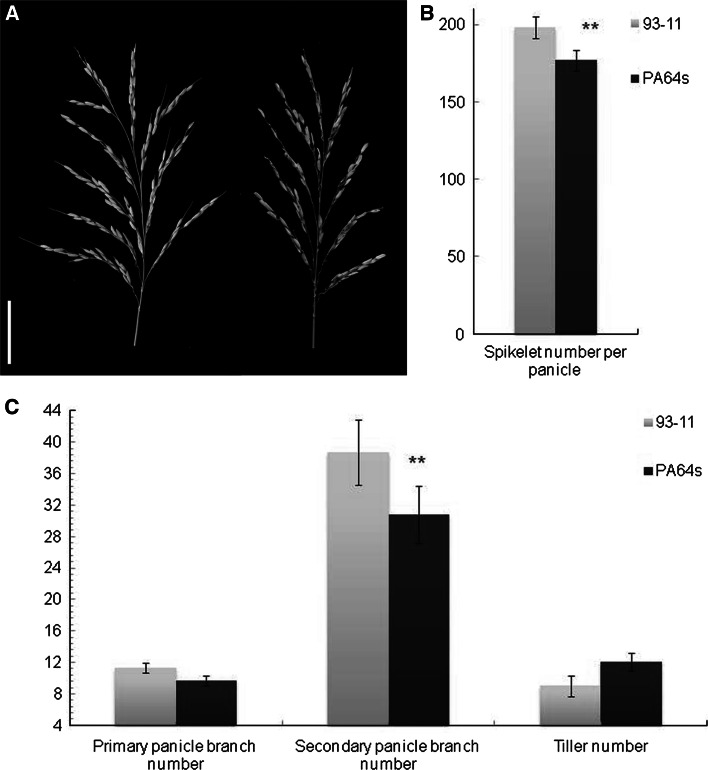
Panicle morphology of *indica* 93-11 and *Javonica* PA64s. **a** Panicle structure of 93-11 (*left*) and PA64s (*right*), *Bar* = 5 cm. **b** Spikelet number per panicle in two parents. **c** Primary panicle branch number, secondary panicle branch number, and tillers per plant of 93-11 and PA64s. Values are the mean ± SD with five replicates

Rice panicle branch traits include primary panicle branch number (PPB) and secondary panicle branch number (SPB). And they directly affect spikelet number per panicle (SN), a crucial factor for rice grain yield. Our study showed PPB was significantly related to SPB and SN, with correlation coefficient 0.5204 and 0.4062, respectively. SPB was significantly related to SN (*r* = 0.8568) (Table 2). The relationships of PPB, SPB, and SN were positive to each other. Therefore, increase in PPB help improve rice grain yield. In parents, spikelet number per panicle, primary panicle branch number and secondary panicle branch number of 93-11 are larger than PA64s. Significant difference existed between parents in spikelet number per panicle and secondary panicle branch number. On the contrary tiller number exhibits less in 93-11 than PA64s. (Fig. 1).

**Table 2:** Correlation coefficient of branch in RIL population

	PPB	SN
SN	0.5204*	
SPB	0.4062*	0.8568*

* Significant at the level of 1 %

### QTL analysis for PPB, SPB, and SN

SNP markers, covered all 12 chromosomes, were used to construct a high-resolution genetic linkage map with total genetic distance of 1,382 cM and an average distance of 0.53 cM between two adjacent SNP markers, as described in Gao et al. (2013).

**Fig. 2:**
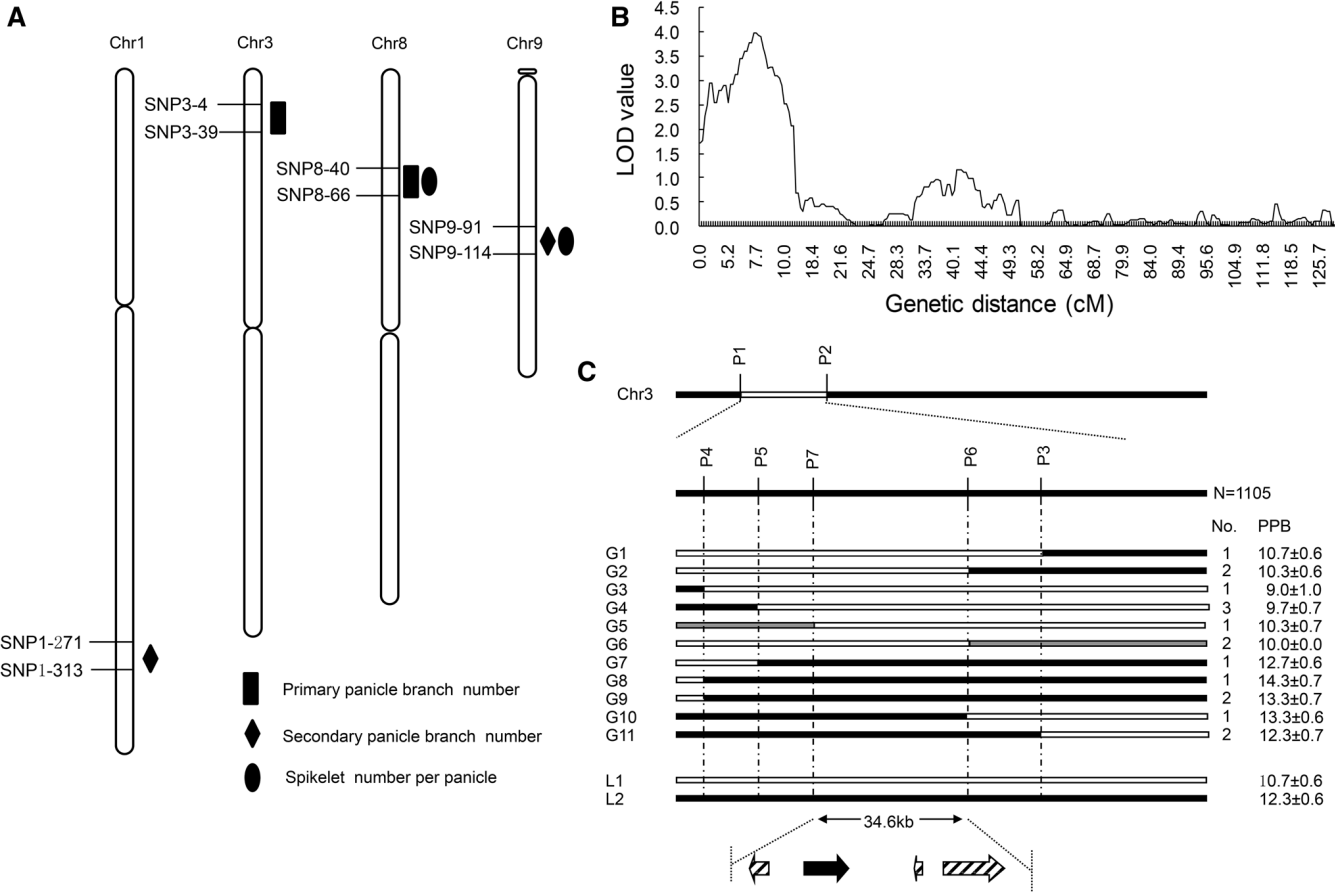
**a** QTLs for panicle traits in the RIL population. SNP markers are shown on the *left* of chromosomes. QTLs signed on chromosomes were detected in several environments and different years. **b** QTL peak map of rice chromosome 3. The genetic distances (cM) are shown below the *x* axis. **c** Fine mapping of *qPPB3*. The *white*, *black*, and *gray* *bars* represent genotypes of PA64s, 93-11, and heterozygote, respectively. All genotypic lines exhibited (G1-G11) were derived from BC_3_F_2_ population

QTL analysis was performed with MultiQTL1.6 using the maximum likelihood interval mapping approach with an LOD threshold 2.5. We detected two QTLs for PPB on chromosomes 3 and 8, named *qPPB3* and *qPPB8*. The *qPPB3* explained 9.4 % phenotypic variation with additive effect came from PA64s. And *qPPB8* explained 15.3 % phenotypic variation came from 93-11. Two QTLs for SPB were identified. The *qSPB1* was mapped on chromosome 1 and explained 10 % phenotypic variation with additive effect came from 93-11. The positive effect of *qSPB9* was from 93-11, explained 9.6 % phenotypic variation. PPB and SPB are two key factors for SN determined by panicle architecture. Two QTLs, *qSN8* and *qSN9* for SN were mapped on chromosomes 8 and 9 with additive effects of 35.5 and 33.2, respectively (Table 3; Fig. 2a). They totally explained 25.4 % phenotypic variation.

**Table 3:** QTL identified for panicle branch in the RIL population

Trait	QTL	Chr.	Pos (cM)	LOD	*P* value	PVE	*A*
PPB	*qPPB3*	3	2.55–12.60	4.00	0.01	0.094	−0.986
PPB	*qPPB8*	8	23.10–28.30	6.03	0.01	0.153	1.258
SPB	*qSPB1*	1	115.40–125.75	2.70	0.01	0.100	3.489
SPB	*qSPB9*	9	31.01–38.34	2.57	0.03	0.096	6.700
SN	*qSN8*	8	23.10–30.45	3.97	0.01	0.136	35.500
SN	*qSN9*	9	30.43–38.53	3.75	0.01	0.118	33.200

### Fine mapping of *qPPB3*

To narrow down the region of *qPPB3*, we developed seven markers (P1–P7). With 1,522 RIL populations, *qPPB3* was fine mapped in the region between markers P1 and P2. Total 1,105 BC_3_F_2_ individuals were utilized to further mapping, and 17 lines were selected from BC_3_F_2_ progenies derived from the cross between GH18 and 93-11. Parent 93-11 and GH18 were designated as the controls L1 and L2, respectively. Out of ten lines with high PPB similar to L2 (genotype of PA64s), there were five recombinant events between P4 and *qPPB3*, four between P5 and *qPPB3*, one between P7 and *qPPB3*, one between P6 and *qPPB3*, four between P3 and *qPPB3*. On the other hand, among seven lines with low PPB similar to L1 (genotype of 93-11), there were two recombinant events between P4 and *qPPB3*, one between P5 and *qPPB3*, one between P3 and *qPPB3*, and P6 and P7 co-segregated with *qPPB3*. Finally, *qPPB3* was delimited to an interval of 34.6-kb region between markers P6 and P7 on the BAC clone AC146702 (Fig. 2b, c).

### A candidate gene for *qPPB3*

**Fig. 3:**
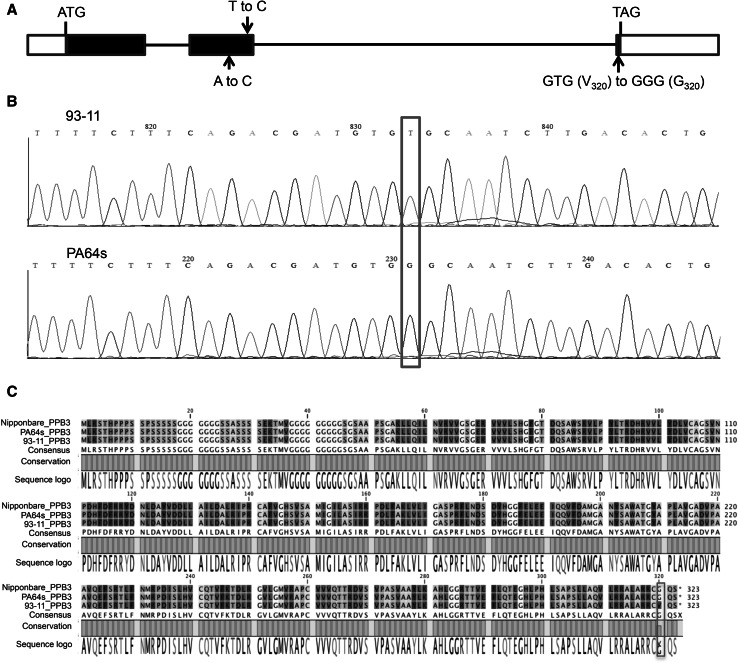
Comparison of genomic sequences and amino acid sequences of rice *qPPB3* between two parents. **a** The *qPPB3* gene is composed of three exons and two introns. *Black* and *white boxes* indicate coding and untranslated region (UTR), respectively, and lines represent introns. *Arrows* indicate the locations of the 93-11 mutation. **b** The nucleotide substitution for change of amino acid is marked with a *red box*. **c** Multiple deduced amino acid sequence alignment of protein coded by *qPPB3* of Nipponbare, 93-11, and PA64s. A *red box* indicates the altered amino acid

To screen candidate genes in the critical 34.6-kb genomic region of Nipponbare genome (http://rapdb.dna.affrc.go.jp/), and four corresponding genes (Os03g0203100, Os03g0203200, Os03g0203700, Os03g0203800) were found. After comparing sequence of the region between parents, only two genes, Os03g0203200 and Os03g0203700, had three SNPs and one InDel between two parents, respectively. However, product of Os03g0203700 was similar to Calcium-transporting ATPase 2, which has been found irrelevant to plant branching so far, and its expression showed no significant difference between two parents. Instead, Os03g0203200 (*D88*) encoded esterase D14/D88 homologous to the α/β-fold hydrolase superfamily protein, which has been reported affecting plant branching and significant difference in its expression level was detected here between 93-11 and PA64s. Only one substitution (T_959_ to G_959_) resulted in amino acid change from valine in 93-11 to glycine in PA64s (Fig. 3). Therefore, Os03g0203200 (*D88*) gene was suggested to be a candidate gene for *qPPB3*.

### Expression comparison of *D88* and panicle structure related genes in initial inflorescence of parents

**Fig. 4:**
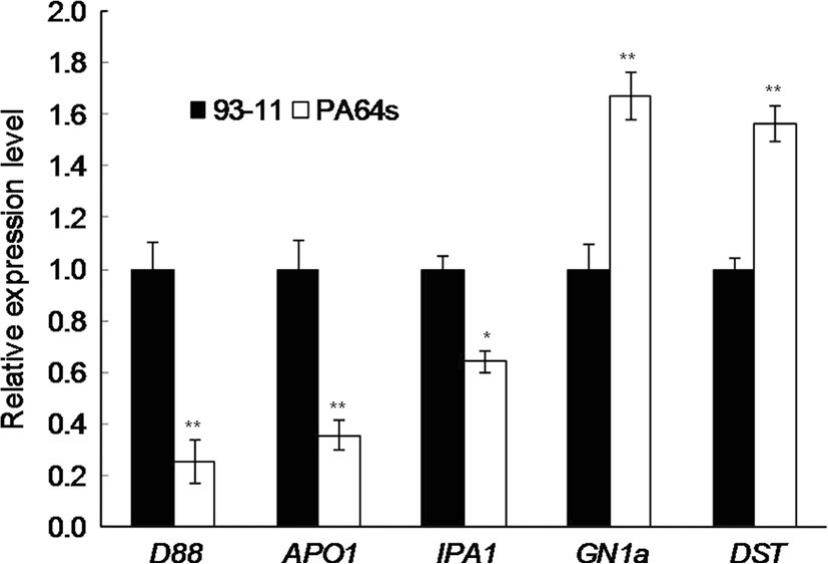
Expression level of *D88* and other panicle related gene by real-time PCR. RNA was isolated from panicle (panicle length <5 mm). * and ** indicate the least significant difference at 0.05 and 0.01 probability level between parents, respectively. Values are the mean ± SD with three replicates

RNA was extracted from inflorescences with length less than 5 mm at the formation stage of panicle primary branch. At the initial formation stage of panicle, the expression level of *D88* in PA64s was significantly lower than that in 93-11 (Fig. 4), which is consistent with the previous report that lower expression of *D88* gene led to higher tillers but smaller panicle in the *d88* mutant (Gao et al. 2009). With the same material, the expression of *APO1* and *IPA1* in 93-11 also significantly increased compared with PA64s, while the expression of *GN1a* and *DST*, two negative regulators of grain number, enhanced in PA64s significantly.

### Subcellular localization of D88

**Fig. 5:**
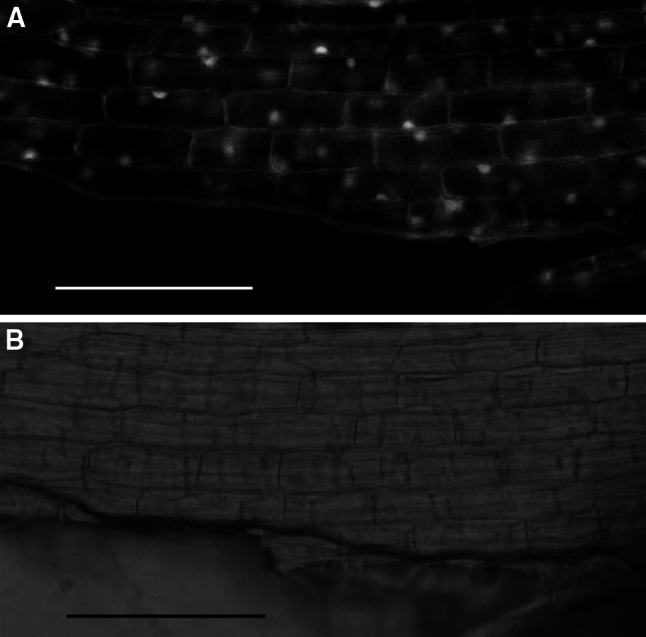
Subcellular localization of D88::GFP fusion protein in rice root cells. **a**, **b** Fluorescent micrographs and light of rice root cells with D88::GFP localization pattern. *Scale bar* = 100 μm

To define the intracellular localization of D88 in rice cells, we introduced D88::GFP into rice by using Agrobacterium transformation. We found that D88::GFP fusion protein was localized to both nucleus and cytoplasm in rice root cells, weakly appeared in the plasma membrane (Fig. 5).

## Discussion

Panicle structure is one of the most important factors for rice yield. To date, 54 QTLs for PPB and 33 QTLs for SPB have been reported distributed on 12 chromosomes (Supplemental Fig. 1). Here, two QTLs were mapped for SPB, *qSPB1* and *qSPB9*. Two QTLs, *sbr1.1* and *qSNB1*-*1* for SPB on chromosome 1 were mapped to the same interval of *qSPB1* (Li et al. 2006; Cui et al. 2002). The *qSPB9* was also reported previously (Li et al. 2006), and *qSN9* for SN was found in the region of *qSPB9*. In this study, two QTLs for PPB, *qPPB3* and *qPPB8* were located on chromosome 3 and chromosome 8, respectively. The region of *qPPB8* was also reported to be related to the QTLs for PPB and SN in other studies (Lin et al. 1996; Xu et al. 2001). One QTL (*QPbn3a*) for PPB and another QTL (*QSbn3a*) for SPB were detected in the same genomic region of *qPPB3* (Xu et al. 2001). The *qPPB3* was further mapped to a region of 34.6-kb on a BAC clone AC146702, where four candidate genes (Os03g0203100, Os03g0203200, Os03g0203700, Os03g0203800) existed. The translational product of Os03g0203200 was D88/D14, an esterase that confirmed to control rice tillering, with *d88/d14* mutants exhibits high tillers but dwarfism (Arite et al. 2009; Gao et al. 2009).

Panicle primary branches developed from primary branch meristems, which are produced by inflorescence meristem. During reproductive development, inflorescence branching is under genetic control affected by hormones that include brassinosteroid (BR), cytokinin (CK), auxin and strigolactone (SL) (Beveridge 2006; McSteen and Leyser 2005; Ongaro and Leyser 2008; Dun et al. 2009; Wang et al. 2013). Like strigolactones insensitive mutants, PA64s also exhibited more tillers and less panicle branches than 93-11. In the study, *qPPB3* was fine mapped to a region covering the *D88* gene, whose product believed to be an important receptor in strigolactone signaling (Jiang et al. 2013). Strigolactones or related compounds were reported to inhibit shoot branching in plants (Gomez-Roldan et al. 2008). At the initial development stage of panicle, the expression level of *D88* was significantly higher in 93-11 than PA64s. And the PPB of 93-11 was larger than PA64s. Therefore, strigolactones or their derivants might be the receptible hormone by D88 in cytoplasm or nucleus to affect the PPB, though regulatory mechanism of branching in panicle is different from that in shoot. In contrast to SL, CK directly promote bud growth and cell proliferation. We tested the expression of two CK related genes, *GN1a/OsCKX2* and *DST*. The expression level of *GN1a/OsCKX2* was significantly lower in 93-11 with more SN, suggesting CK might be accumulated higher in 93-11 than PA64s. Expression level of *DST* was also higher in PA64s, which in accord with its direct regulation of *GN1a/OsCKX2* to control CK level in the reproductive SAM and, as a result, affects panicle branching and grain number (Li et al. 2013).

The *APO1/SCM2*, *GN1a/OsCKX2* and *IPA1/OsSPL14* were cloned QTLs associated with panicle structure. The line contained *APO1* ORF and the proximal promoter region controlled only the number of PPB but not the number of grains per panicle. However, the line included only the distal promoter region that could increase the grain number and harvest index (Terao et al. 2010). The present study also confirmed that the higher PPB in 93-11 exhibited higher expression level of *APO1*. *IPA1* was considered important not only for plant architecture but also for panicle structure. Higher expression of *IPA1/OsSPL14* at the reproductive stage promoting panicle branching is consistent with our results (Miura et al. 2010; Jiao et al. 2010).

Dense panicle structure has been a major target for improvement of rice grain yield because of its relationship with grain number. The utilization of QTLs panicle structure, such as *dep1*, *ipa1*, and *apo1* has increased the crop productivity. Therefore, pyramiding of these elite QTLs, including *qPPB3* mapped here, will certainly benefit high-yield breeding by marker-assisted selection (MAS) for rice in the future.

## Electronic supplementary material

Below is the link to the electronic supplementary material.

Supplementary material 1 (PPT 150 kb)

## Abbreviations

*APO1*: *Aberrant panicle organization 1*
BAC: Bacterial artificial chromosome
BR: Brassinosteroid
CK: Cytokinin
CSSL: Chromosome segment substitution line
*DEP1*: *Dense erect panicle 1*
*DST*: *Drought and salt tolerance*
GFP: Green fluorescence protein
*GN1a*: *Grain number 1 a*
*IPA1*: *Ideal plant architecture 1*
*LAX1, LAX2*: *Lax panicle 1, lax panicle 2*
LYP9: Liang-you-pei-jiu
MAS: Marker-assisted selection
*Moc1*: *Mono-culm 1*
ORF: Open reading frame
*OsCKX2*: *Cytokinin oxidase 2*
*OsSPL14*: *Squamosa promoter binding protein-like 14*
PPB: Primary panicle branch number
qPCR: Quantitative PCR
QTL: Quantitative trait locus
RIL: Recombinant inbred line
SL: Strigolactone
SN: Spikelet number per panicle
*SP1*: *Small panicle 1*
SPB: Secondary panicle branch number
