# Supplementary material for: Fine mapping and candidate gene analysis of a major QTL for panicle structure in rice
Source: Plant Cell Rep. 2014 Jul 31;33(11):1843–50. doi: 10.1007/s00299-014-1661-0 (PMC4197378; doi:10.1007/s00299-014-1661-0)
Supplement: Supplementary file 1 — Supplementary material 1 (PPT 150 kb) [file 299_2014_1661_MOESM1_ESM.ppt]

## Slide 1
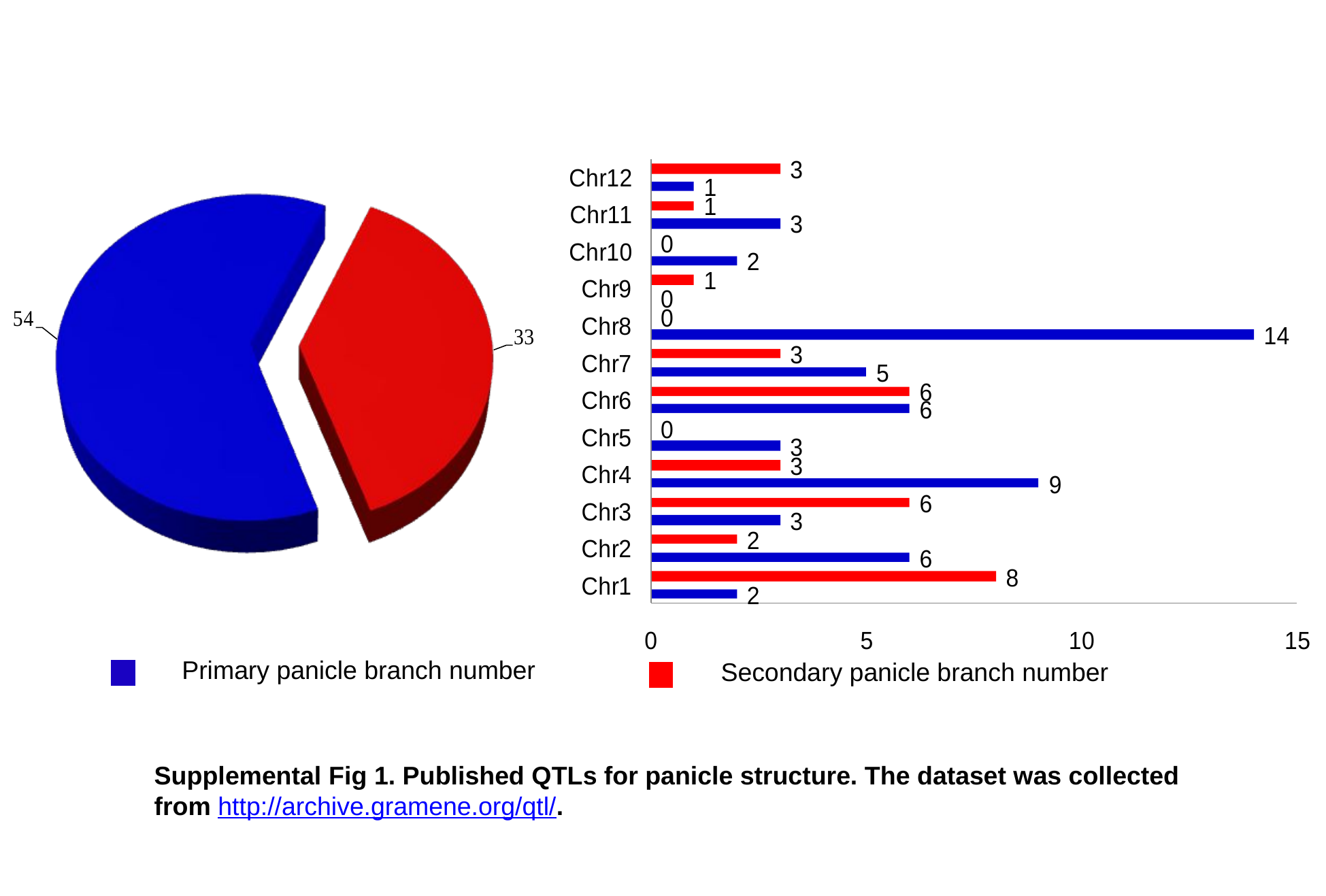

Primary panicle branch number
Secondary panicle branch number
Supplemental Fig 1. Published QTLs for panicle structure. The dataset was collected from http://archive.gramene.org/qtl/.
